# Functional outcome of arterial thoracic outlet syndrome treatment

**DOI:** 10.3389/fsurg.2022.1072536

**Published:** 2023-01-16

**Authors:** Robert J. C. M. F. de Kleijn, Ludo Schropp, Jan Westerink, Eline S. van Hattum, Bart-Jeroen Petri, Gert J. de Borst

**Affiliations:** ^1^ Department of Vascular Surgery, University Medical Center, Utrecht, Netherlands; ^2^ Department of Internal Medicine, Isala Hospital, Zwolle, Netherlands

**Keywords:** arterial thoracic outlet syndrome, ATOS, functional outcome, quickDASH, first rib resection

## Abstract

**Introduction:**

The low prevalence of Arterial Thoracic Outlet Syndrome (ATOS) and diffuse symptomatology have resulted in limited data on optimal treatment strategies and long-term outcome. The aim of this study was to report and evaluate a single center experience with the treatment of ATOS including midterm patient reported outcome.

**Methods:**

All patients treated for ATOS from 2004 to 2020 were retrospectively identified. Patients were divided into two groups based on presenting symptoms; ATOX group (Acute arterial occlusion with ischemia) and ATOS group (claudication symptoms). Baseline characteristics and treatment details were extracted from electronic patient files. A telephone survey was conducted to collect patients' follow-up data including a functional disability score using the Quick Disabilities of the Arm, Shoulder and Hand (DASH) questionnaire. The primary endpoint of this study was symptom-free survival. Secondary endpoints were median QuickDASH scores during follow-up, postoperative complications and possible re-interventions.

**Results:**

A total of 20 patients (mean age 44.6 years, median follow-up 50.5 months) were included and divided into two groups (ATOX *N* = 9, ATOS *N* = 11). In the ATOX group, eight patients were primarily treated with catheter directed thrombolysis (CDT; *N* = 5) or surgical thrombectomy (*N* = 3). All patients received staged thoracic outlet decompression surgery (TOD). In the ATOS group, 10 patients primarily received TOD and one patient was treated conservatively with physiotherapy. Seven ATOX patients and nine ATOS patients were symptom free at follow-up with a median QuickDASH score of 2.3 (IQR 12.5) and 2.3 (IQR 16.5) respectively. Ten complications occurred in the ATOX group; three bleeding complications, five re-occlusions, one arterial dissection and one occipital infarction. In the ATOS group five complications occurred; one perioperative bleeding complication, three re-occlusions and a stent fracture. Seven vs. five re-interventions were required in the ATOX and ATOS groups respectively.

**Conclusion:**

The mid-term self-reported symptom free survival in both the ATOX as well as ATOS group seems acceptable while median QuickDASH scores in both groups indicate a very good functional outcome. This however comes at the cost of treatment related bleeding complications in especially the ATOX group presumably due to thrombolysis, and re-interventions required in almost one out of three patients.

## Introduction

Arterial Thoracic Outlet Syndrome (ATOS) comprises approximately only 1%–2% of all thoracic outlet syndrome patients (1). It is characterized by compression of the subclavian artery between the rigid structures of the thoracic outlet (TO) and associated with a wide range of symptoms. Symptoms vary from chronic arm claudication to acute upper limb ischemia (2). Arterial occlusion in case of ATOS can result from a local progressive stenosis in the subclavian artery or from thrombo-embolization to the distal arteries due to post stenotic aneurysmatic dilatation. In cases with upper extremity ischemia swift restoration of the arterial circulation through catheter directed thrombolysis (CDT) or surgical thrombectomy is required after which staged thoracic outlet decompression surgery (TOD) can be performed to prevent future thrombo-embolic (TE) complications. In ATOS patients experiencing claudication symptoms, only TOD is required to release the subclavian artery and improve circulation to the upper limb. In the acute occlusion subgroup, treatment related bleeding complications, TE complications, and ischemia related residual symptoms are more likely to occur than in the claudication subgroup. Due to the low prevalence of ATOS, only small case series on the treatment of ATOS have been published (3–8). Furthermore, several reported series should be considered outdated due to the ongoing improvements of diagnostic and endovascular treatment options available today (6, 8). As such, there is little data on optimal treatment strategies and long term functional outcome available. The aim of the present study was to report and evaluate both procedural and longer term functional outcome after treatment of ATOS.

## Methods

From 2004 to 2020, all patients referred to our tertiary vascular referral center in the University Medical Center Utrecht suspected of ATOS were retrospectively identified using diagnosis-treatment codes. All patients who met the ATOS diagnosis criteria established by the Society for Vascular Surgery, were included in this study (2). In short, all included patients 1) had chronic claudication symptoms (ATOS group) or acute arterial occlusion of the upper limb (ATOX group), 2) underwent a conventional angiography, MR-angiography or CT-angiography confirming positional subclavian artery compression with or without additional injury to the subclavian artery. Every patient was discussed in a multidisciplinary team specialized in TOS to determine treatment strategy. In our center the preferred method for thoracic outlet decompression surgery (TOD) is *via* a transaxillary approach with a first and/or cervical rib resection combined with scalenectomy. When vascular reconstructive surgery of a subclavian aneurysm is required, a supraclavicular or paraclavicular approach is used. Subclavian aneurysms where defined as a >1.5 × increase in artery diameter. All symptomatic aneurysms where treated simultaneously with TOD, whereas asymptomatic aneurysms with <100% increase in diameter where left untreated.

Patient characteristics, diagnostic imaging, surgical reports, and follow-up data were extracted from patients' electronic medical chart. As part of the follow up, a telephone survey was conducted in which patients were asked about their current symptoms, use of antithrombotic medication, and occurrence of adverse events since treatment. To quantify functional disability the Quick disabilities of the arm, shoulder and hand (DASH) questionnaire was recorded in all patients (9, 10). The QuickDASH is a questionnaire with 11 questions developed to grade the difficulty of performing certain daily tasks and the severity of symptoms of a patients arm on a 5 point Likert scale. The lowest and best score possible being 0, and the worst score 100.

The primary endpoint of this study was symptom-free survival at last follow-up as reported by the patient either at their last hospital visit or in the telephone survey. Secondary endpoints were median QuickDASH scores at last follow-up, number of re-interventions, early (<30 days postintervention), and late complications (>30 days postintervention). Complications included arterial occlusions, bleeding complications according to the World Health Organization (WHO) bleeding score, and treatment related complications (11).

Explorative and descriptive statistical analysis were performed using Statistical Package for the Social Sciences (SPSS; version 25 IBM, Armonk, NY, United States).

## Results

A total of 20 patients were included in this study. The mean age at the time of treatment was 44.6 years (sd 9.9 years), the majority was female (57%), and all patients were classified ASA 1 or 2. The median follow-up was 50.5 months (Range 7–198 months) and 17 out of 20 patients completed the survey including the QuickDASH questionnaire. In 10 patients subclavian artery compression was caused by a cervical rib, in four patients by a fusion of the first and second rib, and in the remaining six patients the compression occurred in an otherwise anatomically normal but narrow thoracic outlet. Initial symptoms at presentation were divided into two groups; patients with acute upper limb ischemia caused by an arterial occlusion (ATOX group, *N* = 9) and patients with chronic arm claudication symptoms (ATOS group, *N* = 11) (Table 1).

**Table 1:** Baseline characteristics.

ATOS patients	*N* = 20
Sex (% male)	9 (45%)
Mean age (years, sd)	44.6 (9.9)
Median follow-up (mos, range)	50.5 (7–198)
**Affected side**	
– Right	10
– Left	10
Dominant side affected (%)	9 (45%)
**Symptom classification (%)**	
– Chronic claudication symptoms (ATOS)	11 (55%)
– Acute arterial occlusion/ischemia (ATOX)	9 (45%)
**Occlusion site (ATOX group only)**	
1. Subclavian artery	2
2. Brachial artery	4
3. Ulnar/radial artery	2
4. Palmar arch/digital arteries	1
**Nature of compression (%)**	
– Cervical rib	10 (50%)
– Fused 1st-2nd rib	4 (20.0%)
– No anatomical variant	6 (30%)
Post dilatation aneurysm (%)	7 (35%)

**Legend:** mos: months, sd: standard deviation.

### Primary treatment

In the ATOX group, initial treatment focused on restoring arterial circulation of the arm. In eight out of the nine patients arterial circulation was successfully restored through CDT (*N* = 5) or surgical thrombectomy (*N* = 3) depending on the location and extend of the occlusion. (see Table 1, occlusion sites) In the one patient with multiple occlusions of the digital arteries and palmar arch as a result of distal trashing, CDT was considered technically impossible. In all nine patients TOD was performed in a semi-elective setting; six *via* transaxillary approach and three *via* supraclavicular approach. In two patients a post stenotic aneurysm with fresh intraluminal thrombus was found as embolus source and a third aneurysm was found without intraluminal thrombus. Both symptomatic aneurysms were treated; one with a Polytetrafluoroethylene (PTFE) interposition bypass and the second by resection of the aneurysm with a direct end-to-end anastomosis. In the third patient the aneurysm was primarily left untreated, but required endovascular stenting after a dissection occurred (Table 2).

**Table 2:** Treatments, and symptom free survival.

	Group 1 (*N* = 9)	Group 2 (*N* = 11)
**Primary treatment**		
1. TOD only	1	9
2. CDT + staged TOD	5	–
3. Surgical thrombectomy + staged TOD	3	–
4. TOD + primary stenting	–	1
5. Mensendieck physical therapy	–	1
**Postoperative antithrombotic treatment**		
1. ASA	2	3
2. Clopidogrel	1	3
3. DAPT	1	1
4. Vitamin K antagonist	5	–
5. DOAC	–	1
6. None	–	3
Symptom free at last follow-up	7 (78%)	9 (82%)
QuickDASH score (median, IQR)	2.3 (12.5)	2.3 (16.5)

**Legend:** ASA: acetylsalicylic acid, CDT: catheter directed thrombolysis, DAPT: dual antiplatelet therapy, DASH: disabilities of the arm, shoulder and hand, DOAC: direct oral anticoagulant, IQR: interquartile range, TOD: thoracic outlet decompression.

In the ATOS group, 10 out of 11 patients were primarily treated with TOD; 10 *via* transaxillary approach and one *via* supraclavicular approach because of a prior radical mastectomy on the affected side. Two patients received additional percutaneous transluminal angioplasty (PTA) and one required additional postoperative stenting for a PTA resistant stenosis. One patient was treated conservatively with physiotherapy focused on posture improvement. Post stenotic aneurysms where found in four patients and all were treated conservatively without leading to TE complications during follow-up.

### Postoperative antithrombotics

A total of 17 patients received postoperative antithrombotic therapy. In the ATOX group, all nine patients received postoperative antithrombotic therapy; acetylsalicylic acid (ASA) (*N* = 2), clopidogrel (*N* = 1), dual antiplatelet therapy (DAPT) (*N* = 1) and vitamin K antagonists (*N* = 5). (Table 2) Therapy duration varied between three (*N* = 2) to four months (*N* = 1) and lifelong antithrombotic therapy (*N* = 6).

In the ATOS group, eight out of 11 patients, including the stented patients, received lifelong antithrombotic therapy while 3 patients received no postoperative antithrombotics whatsoever. Prescribed were ASA (*N* = 3), clopidogrel (*N* = 3), DAPT (*N* = 1), and Direct Oral Anticoagulants (DOAC) (*N* = 1).

### Symptom free survival

After a median follow-up of 50.5 months (range 7–198 months) a total of 16 patients (80%) reported to be symptom free at last follow-up. This was confirmed by the median QuickDASH scores of the whole cohort (2.3, IQR 12.5) and ATOX (2.3, IQR 12.5) and ATOS subgroups (2.3, IQR 16.5). (Table 2) In the ATOX group two patients were symptomatic at last follow-up (at 92 and 198 months respectively) experiencing claudication symptoms during exercise, with QuickDASH scores of 36.4 and 18.2 respectively. In the ATOX group, none of the patients experienced long term residual symptoms related specifically to their ischemic event.

In the ATOS group one patient suffered from venous TOS on the contralateral side and underwent TOD. After 61 months of follow-up she remained symptomatic on both sides with a QuickDASH of 95.5. No clear substrate for her symptoms could be found despite extensive diagnostic analysis. The other symptomatic patient reported sporadic fatigue after intensive overhead labour after a follow-up of 24 months with a QuickDASH score of 20.5.

### Complications and re-interventions

A total of seven early complications occurred. In the ATOX group two puncture site bleedings after CDT (grade 2) occurred and one haematothorax (grade 3) that required coiling of the internal mammary artery. During this coiling procedure a dissection of an already known subclavian artery aneurysm was discovered and stented with self-expendable stents. This patient developed an ipsilateral occipital infarction with hemianopia three days later. On diagnostic imaging no clear embolus source was discovered. Finally, one patient suffered an early recurrent occlusion in-between surgical thrombectomy and TOD, which was successfully treated with CDT. In the ATOS group one patient developed a haematothorax after TOD that required thorax drainage.

Eight complications occurred over 30 days after initial treatment. Two patients in the ATOX and two in the ATOS group suffered a total of seven recurrent occlusions (ATOX group *N* = 4, ATOS group *N* = 3). All occlusions occurred while under antithrombotic therapy (three ASA, one DAPT), and three out of seven were in-stent occlusions. In the ATOS group one stent fracture occurred over an insufficiently resected first rib (Table 3).

**Table 3:** Complications and re-interventions.

	ATOX group (*N* = 9)	ATOS group (*N* = 11)
**Complications <30 days**		
1. Re-occlusion	1	–
2. Bleeding complication*		
a. Grade 1	0	0
b. Grade 2	2	0
c. Grade 3	1	1
d. Grade 4	0	0
3. CVA	1	–
4. Arterial dissection	1	–
Total (number of affected patients):	6 (4)	1
**Complications >30 days**		
1. Re-occlusion	4 (2)	3 (2)
2. Stent fracture	–	1
Total (number of affected patients):	4 (2)	4 (3)
**Re-interventions**		
1. CDT	4	1
2. CDT + stent	1	–
3. TOD	–	1
4. Re-resection first rib	1	–
5. PTA	–	1
6. Stent placement	1	2
Total (number of affected patients):	7 (3)	5 (3)

**Legend:** CDT: catheter directed thrombolysis, CVA: Cerebrovascular Accident. PTA: percutaneous transluminal angioplasty, TOD: thoracic outlet decompression.

* WHO bleeds.

Six patients required a total of 12 re-interventions during follow-up. In the ATOX group, three patients required a further four CDT interventions, one CDT intervention with stent placement in the brachial artery for a significant PTA resistant stenosis over 40 mm, one redo-resection of an insufficiently removed and regrown first rib causing residual symptoms and one subclavian artery stent placement in a patient with recurrent claudication symptoms. In the ATOS group three patients required one CDT intervention, one PTA, one stent-in-stent placement to treat a stent fracture, one primary stent placements for PTA resistant stenosis with recurrent claudication symptoms and finally a TOD in the primarily conservatively treated patient to resolve her claudication symptoms (Table 3).

## Discussion

This study shows that adequate surgical and endovascular treatment of ATOS can lead to almost full symptom resolution and an acceptable symptom-free survival after a median follow-up of more than four years. However, early bleeding complications and late recurrent arterial occlusions are not uncommon and re-interventions are required in almost one in three treated patients.

This study is one of the larger case series on ATOS (3–5, 8). However, the long median follow-up of more than four years in combination with the recorded QuickDASH scores at last follow-up has been rarely reported and allows for a better understanding of the mid- to long-term morbidity of ATOS. The use of the QuickDASH-score has been advocated by experts in a recent Delphi study as the best suited functional disability score to determine functional outcome in patients with upper extremity post thrombotic syndrome (UE-PTS) after VTOS (12, 13). We believe that this score has equal merit in assessing the functional disability in ATOS patients since symptoms (e.g., fatigue, pain, loss of strength and function of the arm) are similar in both ATOS patients as well as patients with UE-PTS. Compared to other case series, we achieved similar treatment results with a symptom free survival in 80% of our patients. Moreover, we were able to compare their symptom free status with the QuickDASH scores reported at last follow-up. With a median QuickDASH score of 2.3 this indicates an almost complete recovery in the majority of our patients.

Compared to other series we used a less invasive approach regarding subclavian artery reconstruction surgery (3, 4, 7). In this cohort only two out of seven patients with a post stenotic aneurysm underwent surgical arterial reconstruction and one dissection of a subclavian aneurysm was stented (all in the ATOX group). The other four patients with aneurysms suffered only from chronic claudication symptoms (ATOS group) and were left untreated without causing TE complications during follow-up. This might suggest that it is safe to not perform subclavian artery reconstruction in patients with mild post stenotic aneurysms without TE complications (5).

In this series, a total of five stents were placed in four patients after TOD with mixed results. This seems in contrast with the most recent ESVS guidelines discouraging stent placement for TOS treatment. However, we would like the emphasize that in half of these patients the indications for stenting were not related to TOS treatment (subclavian artery dissection *N* = 1) and/or were not placed in the thoracic outlet (brachial artery stent *N* = 1). Nonetheless, with the currently limited data available, we believe that stenting in the thoracic outlet should be applied cautiously and be considered a last resort option in patients with severe claudication symptoms (14).

No treatment protocol for postoperative antithrombotic therapy in ATOS patients exists. This was reflected in the variety of prescribed antithrombotics. All seven recurrent occlusions that occurred during follow-up, transpired in patients who were put on antiplatelet therapy, while none occurred in the patients using vitamin K antagonists or DOACs which might suggest that antiplatelet therapy is an inadequate therapy for postoperative ATOS patients. However, three out of seven re-occlusions occurred in patients with subclavian artery stents known for an increased risk of recurrent TE events and therefore an important confounder (by indication) in this study (15). Based on our series and currently available evidence an optimal postoperative antithrombotic treatment strategy for prevention of recurrent arterial occlusions in ATOS patients is still lacking. Hence, future research in prospective registries, such as the TROTS registry, is warranted (16).

This study has several limitations. We only performed postoperative imaging on indication, including patients with conservatively treated post stenotic aneurysms, and were therefore unable to assess the postoperative arterial patency over time. However, we believe that symptom status and functional outcome measures are more insightful and relevant for the patient than standardized arterial patency assessment. Even for the patients with conservatively treated post dilatation aneurysms, standardized imaging might not be strictly necessary since none of these patients experienced TE complications in our series so far. Moreover, in a recent series that performed standardized follow-up imaging conservatively treated post stenotic aneurysms in ATOS patients, no further growth of the aneurysms or vascular complications were found (5). We therefore believe that standardized imaging in the long-term follow-up in ATOS patients is of little clinical added value.

Further limitations are the retrospective nature and small sample size. However, due to the low prevalence of ATOS, large prospective case series are hard to come by and can only be achieved through multicenter prospective registries. Despite these limitations, this study shows that the mid- to long term prognosis of ATOS seems favorable after adequate surgical and endovascular treatment. With the start of our multicenter TROTS registry on the treatment of thoracic outlet syndromes we will be able to expend and update this case series with prospectively collected data in the future.

## Conclusion

The mid-term self-reported symptom free survival in both the ATOX as well as the ATOS group seems acceptable and the median QuickDASH scores in both groups are very good. However, this comes at the cost of treatment related bleeding complications in especially the ATOX group, recurrent arterial occlusions and re-interventions were required in almost one of every three treated patients.

## Data Availability

The raw data supporting the conclusions of this article will be made available by the authors, without undue reservation.
